# PS-10 - MASS CASUALTY PLANS FOR A RESILIENT HEALTH AND SOCIAL CARE SYSTEM: WHAT RISKS AND IMPACTS DO PLANNERS CONSIDER

**DOI:** 10.1093/pubmed/fdag046.059

**Published:** 2026-07-09

**Authors:** Dr Padraig Lyons, Professor Kate Cochrane

**Affiliations:** NHS Highland; NHS Highland

## Abstract

**Background:**

Mass casualty incidents (MCIs) such as major accidents, fires, or other natural disasters present multifaceted challenges that can rapidly overwhelm health and social care systems. Existing service pressures within health and social care services raise concerns about the adaptability and sustainability of current mass casualty planning approaches, including how recovery is addressed.

**Objectives:**

This study aimed to explore how mass casualty planning and incident response are undertaken across the UK, identify key facilitators and challenges influencing preparedness, examine approaches to exercising mass casualty plans, and understand how recovery is incorporated into planning and response frameworks.

**Methods:**

In this qualitative study, 12 semi-structured interviews were conducted with professionals involved in mass casualty planning or incident response across England and Scotland. Participants were purposively sampled from Integrated Care Boards, Local Resilience Forums, public health departments and acute healthcare services. Data were analysed using thematic analysis.

**Results:**

Four primary themes were identified which illustrate the different strategies adopted when planning for MCI’s as well as the challenges involved in responding to incidents. Effective collaboration and communication within and across organisations were identified as key facilitators, supported by clear, pragmatic plans. Key challenges included chronic NHS capacity pressures, workforce constraints, frequent organisational change, geographical variation, and difficulties translating national risk assumptions to local contexts. Regularly exercising plans was universally viewed as essential but limited by resource constraints. Recovery was recognised as important but inconsistently planned and under-resourced, particularly regarding long-term psychological impacts on staff and impacted communities.

**Conclusions:**

Effective MCI response depends on sustained collaboration across all stages of planning. Co-production of plans and strong cross-organisational working are central to effective incident response, however, resource limitations and organisational pressures can constrain planning and implementation.

